# B cell memory: from generation to reactivation: a multipronged defense wall against pathogens

**DOI:** 10.1038/s41420-024-01889-5

**Published:** 2024-03-07

**Authors:** Madiha Zahra Syeda, Tu Hong, Chunming Huang, Wenhua Huang, Qingchun Mu

**Affiliations:** 1 The People’s Hospital of Gaozhou, Guangdong Medical University, Maoming, 525200 China; 2 School of Basic Medical Sciences, Southern Medical University, Guangzhou, 510515 China; 3 The First Affiliated Hospital, Zhejiang University, School of Medicine, 310058 Hangzhou, China

**Keywords:** Cell division, Immunology, Infectious diseases

## Abstract

Development of B cell memory is a conundrum that scientists are still exploring. Studies have been conducted in vitro and using advanced animal models to elucidate the mechanism underlying the generation of memory B cells (MBCs), the precise roles of MBCs against pathogens, and their protective functions against repeated infections throughout life. Lifelong immunity against invading diseases is mainly the result of overcoming a single infection. This protection is largely mediated by the two main components of B cell memory—MBCs and long-lived plasma cells (PCs). The chemical and cellular mechanisms that encourage fat selection for MBCs or long-lived PCs are an area of active research. Despite the fact that nearly all available vaccinations rely on the capacity to elicit B-cell memory, we have yet to develop successful vaccines that can induce broad-scale protective MBCs against some of the deadliest diseases, including malaria and AIDS. A deeper understanding of the specific cellular and molecular pathways that govern the generation, function, and reactivation of MBCs is critical for overcoming the challenges associated with vaccine development. Here, we reviewed literature on the development of MBCs and their reactivation, interaction with other cell types, strategies against invading pathogens, and function throughout life and discussed the recent advances regarding the key signals and transcription factors which regulate B cell memory and their relevance to the quest for vaccine development.

## Facts

- Terminal differentiation of B cells to Memory fate is a multi-factorial process.
- There is no single master regulator for fate determination in dividing B cells.
- Interaction with T cells play important role towards fate determination in dividing B cells, however, T-cell independent memory B cells are also produced.
- B cell memory elicits a multipronged defense against invading pathogens and repeated infections. The origin, function, and lifetime of MBCs differ among the different subtypes.
- Nearly all available vaccinations rely on the capacity to elicit B-cell memory, we have yet to develop successful vaccines that can induce broad-scale protection against some deadly diseases, including Malaria, and AIDS.

## Open Questions

- What are the main parameters that govern the fate determination in dividing B cells?
- What is a key factor component by which low Tfh cell help favors the differentiation towards memory fate.
- What are the mechanisms underlying heterogeneity in Memory B cells population, and whether distinct MBCs subtypes are coordinately activated after a particular response?
- What are the different strategies that Memory B cells use to fight against a novel pathogen or repeated infection? And how can the B cell Memory be employed to develop successful vaccines against different diseases.

## Introduction

To successfully fight an invading pathogen, B cells must organize a multilayered defense and attack system. They can give rise to antibody-secreting plasma cells (PCs), develop into T cell-assisted germinal center (GC) cells, or even differentiate into long-lived memory B cells (MBCs). Protective antibodies already present in the body and released by long-lived PCs provide a primary defense against reinfection, which is known as constitutive humoral memory. If constitutive memory is insufficient, another defense mechanism, reactive humoral immunity, is activated in which pathogen-experienced MBCs from a previous infection are quickly reactivated to produce antibodies [1]. Previously, it was thought that reactive humoral memory was a backup system for constitutive humoral immunity, especially against homologous infections. Although the response of reactive humoral immunity is faster and of greater amplitude, consisting of isotype-switched antibodies with higher affinity towards identical yet distinct viral strains [2], the increased responsiveness and preservation are frequently attributed to the action of a limited number of MBCs that were produced in the original immune response against the antigens and survived. Therefore, understanding the basis of humoral immunity requires identifying the features of MBCs and determining how these distinct features develop. The efficiency of MBCs was previously thought to originate from the class-switched and high-affinity B cell receptors (BCRs) on their surfaces, which develop within the GCs [3–5]. However, recent studies have clearly revealed the existence of GC-independent MBCs [6–8], as well as unswitched MBCs [9–11].

Here, we summarize the literature and discuss recent advances in the field and associated questions from a traditional MBC biology perspective. We specifically discuss studies in mice, as new elements of MBC development have been discovered in recent years following novel updates in mouse models. Furthermore, we discuss how this information may contribute to the search for vaccines against HIV, influenza, and other pathogenic diseases.

## Generation of MBCs

B cells are activated upon antigen encounter and finally get differentiated into Plasma cell or memory cell. Previously, B2 cells (found in second lymphoid organs (SLOs), and generate antigen specific antibodies) were considered the exclusive participants in the generation of MBCs because of the concept that MBCs are only generated in a T-cell dependent immune response against protein antigens. However, later research showed that B1 cells (predominantly located in peritoneal cavity, and generate natural antibodies) could also develop into MBCs during a T-cell dependent immune response [12–14]. Below, we explain the roles of T-cell dependent and T-cell independent antigens in memory generation.

### Generation of T cell-dependent MBCs

First antigen encounter of the naïve B cells in SLOs stimulates the BCR downstream signaling and directs the internalization of the BCR-bound antigen peptides followed by their processing and presentation on the MHC-II molecules. The B cells that are activated by the antigen have increased metabolic activity and chemoattractant receptors (i.e., CCR7 and EBI2) that guide them to the border of the T cell zone [15]. Here, they interact with antigen specific T helper cells that have been prepared by antigen-presenting follicular dendritic cells (FDCs) to differentiate into T follicular helper (_Tfh_) cells. The close proximity of the naïve B cells and T helper cells facilitates the binding of several surface molecules (TCR-MHC, CD28-B7, CD40-CD40L, and other adhesion molecules) between them [16]. They can then differentiate into one of three fates: short-lived PCs, GC B cells, or MBCs in the follicle, which are independent of GCs. B cells from mice lacking BCL-6, which are unable to produce GCs, demonstrate the ability to differentiate into both IgG^+^ and IgM^+^ MBCs without acquiring somatic hypermutation (SHM), but not into long-lived PCs [17]. MBC generation is primarily studied in the drainage lymph nodes or spleen. The determination of the fate of MBCs in a classical T cell-dependent antibody response can occur at two distinct stages, as discussed below.

### MBC determination at the pre-GC and GC-independent stages

At the T cells–B cells border, the freshly activated B cells can either join a GC or differentiate into PCs or MBCs. Subsequently, within the GCs, they can again either recycle GC cells or differentiate into PCs. Although many pre-GC MBCs have unswitched IgM+ isotype with unmutated and low-affinity BCRs, -switched isotype IgA + /IgG+ MBCs also exist, which could be attributed to isotype switching that occurs early, during the pre-GC stage [7, 8, 18, 19]. Thus, the precise mechanism by which these GC-independent MBCs are generated remains a subject of active research.

### Generation of GC-independent MBCs

**Fig. 1:**
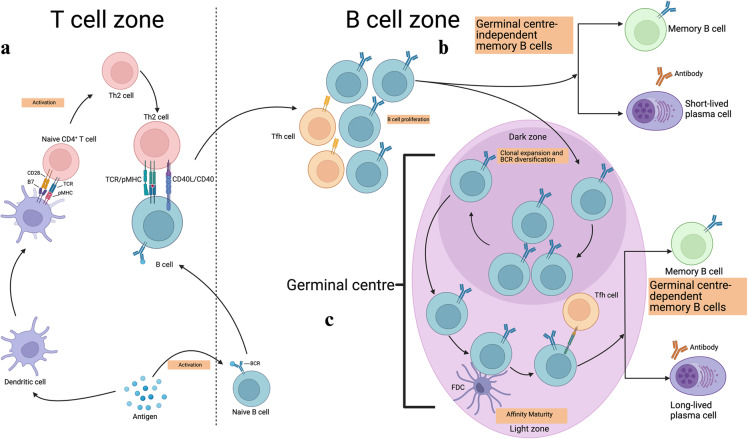
T cell-regulated generation of MBCs. **a** Naïve B and T cells migrate to the border between the B cell follicular and T cell zones after antigen encounter in the secondary lymphoid organs. This allows the B cells to develop stable contact with T cells and receive CD4 + T cells derived helper signals. These antigen activated B cells then move towards the outer follicles where they can proliferate further and choose between three fates. **b** Among these proliferating B cells, some will differentiate into short-lived PCs, while some join the GC-independent memory B cell population. **c** The remaining proliferating B cells reenter the B cell follicle and undergo rapid proliferation, thus establishing a germinal center. Within the dark zone of the germinal center, somatic hypermutation (SHM) diversifies the B cell receptors (BCRs) in the actively proliferating B cells (clonal expansion). Some of these cells relocate to the light zone of the germinal center where they interact with the antigen-presenting follicular dendritic cells and antigen-specific T follicular helper (Tfh) cells to undergo affinity maturation. These affinity-selected B cells can either rejoin the GC cycle or exit the germinal center as terminally differentiated cells, either as long-lived PCs or germinal center-dependent memory B cells. PCs Plasma cells, BCR B cell receptor, TCR T cell receptor. Reproduced with permission.

Research have shown a critical role that various T cells mediated signals play in the generation of MBCs. Among these, CD40-signal individually has the ability to induce the differentiation of activated B-cells into MBCs, but not into GC cells [7]. Cytokine signaling is likely essential for the development of GC B-cells in addition to CD40 signal. Certainly, the interleukin-21 (IL-21) can elevate the BCL-6 level in B-cells, which is a critical transcription factor in the GC development and maintenance [20, 21]. Based on these observations, Tomohiro et al. [1] suggested that, to receive sufficient T cell help, B cells must form durable conjugates with Tfh cells. This allows the B cells to differentiate into GC B cells (Fig. 1a). In contrast, if the conjugate only endures for a relatively brief period, the B cells join the GC-independent MBC pool (Fig. 1b). Because class switching occurs at the early stages (pre-GC stage) but not SHM, the BCR specificities of GC-independent MBCs are expected to be similar to the specificities of early responder B cells, although the cognate T cell help may impose some selection on the GC-independent phases during priming. Thus, unlike the GC-dependent MBCs discussed below, GC-independent MBCs allow the maintenance of a wide variety of antigen-specific B cells that provide protection against related but mutated pathogen antigens. This suggests that GC-independent MBCs may be an adaptation or conservation mechanism that allows B cells to maintain an MBC pool with a broad range of BCR affinities. Because MBCs in the pre-GC phase develop relatively early (probably before pathogen clearance from the body in primary infection), it is likely that these MBCs join the ongoing immune response again at later time points and undergo further BCR diversification. Moreover, these early MBCs could serve as evolutionary templates for mutation and selection in secondary GCs upon exposure to variant strains of a previous infection, allowing them to build high-affinity BCR repertoires for the novel epitopes of these variant strains.

### Generation of T cell-dependent MBCs within the GC

Second stage of memory generation in germinal centers is primarily concerned with the development of B cells with high affinity via selection (Fig. 1c). During this phase, germinal center-B cells that have developed from the naïve B cells now join the GCs; clusters of closely packed cells in the follicles. Adoptive transfer experiments have revealed that MBCs develop from the activated precursor cells that express GL7 and CD38 on their surface [7, 8]. GCs are typically divided into two distinct zones: a dark zone (DZ) and a light zone (LZ). The DZ serves as a primary site for proliferation and SHM, while the LZ serves as the main site for selection. Both BCL-6 (a transcription factor) and the G-protein coupled receptor sphingosine 1-phosphate receptor 2 are highly expressed in GC B cells, facilitating their retention in the GC [22–24]. In the DZ of the GC, GC B cells undergo proliferation and SHM before entering the LZ, where they meet one of the following fates. First, in the absence of an antigen or secondary infection, some GC B cells develop into long-lived PCs that reside in the bone marrow and start secreting antibodies, lasting years or even a lifetime [25, 26]. Second, some of the GC B cells will differentiate to produce long lived MBCs, which quiescently reside in niches within the SLOs, enabling them to be exposed to antigens. Third, instead of terminally differentiating, these GC B cells return to the DZ in GC to undergo further rounds of SHM and selection, eventually differentiating into long lived PCs or MBCs [27–30]. On the LZ GC B cells, two antigen-based signaling systems are present: one via BCR, and the other through cognate interaction with Tfh Cells.

Victora et al. [31] reported that only a fraction (approximately 10–30%) of the cells arriving in the LZ are redirected to join the DZ, while the remaining cells either exit the GC or undergo apoptosis. This implies that the selection of the best LZ GC B cells to re-enter the DZ and leave as PCs is among the main regulatory processes to achieve the affinity maturation. The dynamics of GC reactions have been explored and revealed in several recent studies [32–36] and have been reviewed in detail by De Silva and Klein [37]. Here, we highlighted the mechanistic pathways that direct the development of MBCs from GC B cells. Previously, it was hypothesized that a master regulator of transcription might influence and determine the fate of B cells towards MBC. However, no single fate-determining factor has been identified despite extensive research and gene expression analysis. As a result, a popular alternative theory holds that MBCs differentiate from GC B cells through a stochastic mechanism, and that a survival advantage favors MBC differentiation. The induction of BCL-2 expression (a pro-survival signal) or the deletion of pro-apoptotic factors such as BIM (also called BCL-2L11) or PUMA (also called BBC3) resulted in increase in the size of the IgG+ MBC compartment [38, 39]. In support of this, the expression levels of BCL-2 and BIM in IgG+ MBCs were higher and lower, respectively, than those in activated B cells [8, 40].

### Generation of T cell-independent MBCs

Although peritoneal cavity has most abundant B1 cells, low but discernable frequency can also be detected in the spleen [41]. They are classified as CD5+ (B1a) and CD5− (B1b) cells. B1 cells have also been reported to elicit memory responses, particularly in a T cell-independent manner. For B1a cells, Yang et al. [14] showed that priming with a live *Francisella tularensis* vaccine strain-derived glycolipid (FtL) triggered the development of FtL-specific long-lived memory B1a cells (mostly IgM + ), which persisted only in the peritoneal cavity in a T cell-independent manner. PC differentiation upon rechallenge with FtL required co-stimulation with a TLR4 agonist [14]. It appears that peritoneal cavity-resident memB1a cells migrate to spleen upon rechallenge when differentiated into PCs. These observations indicate the importance of the microenvironment in the maintenance and activation of memB1a cells, which may differ from conventional MBCs. For memB1b cells, tracing the antibody responses to *Borrelia hermsii* and *Streptococcus pneumoniae* infections revealed the generation of memory B1b cells that persisted in the peritoneal cavity, similar to memory B1a cells [12, 42]. More recently, Obukhanych et al. [13] traced and analyzed 4-hydroxy-3-nitrophenylacetic-Ficoll (an antigen)-specific B cells and revealed that memory B1b cells retained the phenotypic characteristics (longevity and sensitivity to antigen stimulation) of naïve B1b cells [13]. These data indicate that primary characteristics of an antigen specific memory B1 cell resemble their parent naïve B cell.

Numerous pathways are involved in MBC development. Although T cell-independent MBCs may be produced, their recall responses appear to be quantitative rather than qualitative. In addition to the higher frequency of antigen-specific B cells, it is unknown if T cell-independent MBCs carry an inherent advantage over their naive (B cell) counterparts in terms of responding promptly and robustly to antigens, similar to T cell-dependent MBCs. We discuss the numerous characteristics and routes of development of canonical T cell-dependent MBCs in the following sections.

## Models of MBC development

**Fig. 2:**
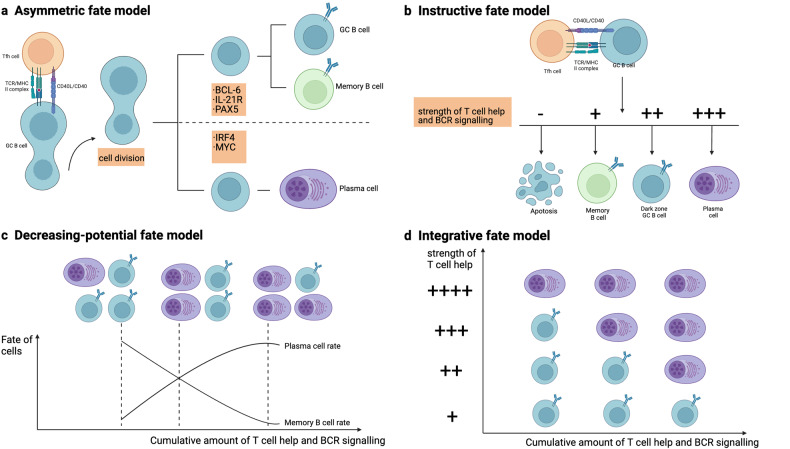
Different models of fate determination in germinal center (GC) B cells. Schematic illustrations of four different models that could be involved in the fate determination process of GC B cells. **a** The asymmetric fate model suggests that interaction with follicular helper T (Tfh) cells induces polarization in GC B cells, which results in the asymmetric division of fate-determining molecules. Daughter cells inheriting BCL-6, IL-21R, and PAX5 will either retain the GC B cell state or join the memory B cells compartment. In contrast, daughter cells receiving MYC and IRF4 undergo differentiation into PCs. **b** In the instructive fate model, the Tfh cell help gained by the GC B cell directs its fate. A strong degree of help favors PC differentiation, whereas weak help from T cells results in either memory fate or apoptosis. **c** In the decreasing potential fate model, a cumulative strength of T cell help and B cell receptor (BCR) signaling over time determines the fate of GC B cells. Repeated proliferation cycles gradually reduce the capability of GC B cells to differentiate into memory B cells, leading to increased plasma cells at later time points in GC reactions. **d** In the integrative fate model, the quality and quantity of T cell help and BCR signaling are all important in fate determination. It explains the preferential differentiation of GC B cells into PCs at late GC time points. MHC Major histocompatibility complex, CD40L CD40 ligand, TCR T cell receptor. Reproduced with permission.

Several models have been proposed to describe MBC development [43, 44], and Laidlaw and Cyster [45] have explained them in great detail. Here we provide a brief overview of these models (Fig. 2).

### Asymmetric fate model

Studies have shown that interactions with Tfh cells induce asymmetric cell division in GC B cells, resulting in an unequal distribution of the molecules that determine cell fate among their progeny [46, 47]. Daughter cells inheriting IRF4 and MYC favorably differentiated into PCs, while cells receiving PAX5, IL-21R, and BCL-6 either retained their identity as GC B cells or differentiated into MBCs (Fig. 2a). However, the functional importance of unequal cell division in regulating the differentiation towards MBC has been challenged by in vitro research using activated B cells and the mathematical modeling based research, which imply that GC B cells typically divide symmetrically [44, 48].

### Instructive fate model

This model suggests that external cell signals, including cytokines (such as IL-9 and IFNγ) and cell contact-dependent signals (such as CD40–C4BP), are involved in final fate determination. Supporting this idea, disruption of the ability to receive T cell assistance favors the increase of MBC population that is disproportionate to the size of germinal center [20, 49, 50]. Antigen affinity is a key factor in this model, as GC B cells with higher-affinity benefit from relatively strong T cell assistance, which encourages differentiation into PCs and DZ states, while weak assistance from T cells favors either the differentiation of GC B cells into memory compartment or apoptosis [44] (Fig. 2b). However, this model fails to explain the common observing that MBCs typically originate from GC B cells before PCs [51–53].

### Decreasing potential fate model

This model emphasizes the total strength of the signals that GC B cells accumulate over time in the GCs (over days to weeks). Since GC B cells perform repetitive cycling, early GC reactions accumulate less T cell help and favor MBC development. As the GC reaction advances, the overall amount of T cell help increases, favoring higher PC differentiation at later time points (Fig. 2c). This model is supported by the transcriptional and functional differences found in GC B cells at early and late time points. However, the specific mechanisms underlying this model are unclear.

### Integrative fate model

According to this model, when deciding cell fate, the current quality and overall strength of the signals received are integrated by the GC B cells. This suggests that even if GC B cells have a history of signaling that predisposes them to particular fates, quality of the T cell assistance gained prior to differentiation still affects the final cell fate (Fig. 2d). To develop into PCs, GC B cells receiving comparatively weak overall T cell assistance and BCRs signaling require a relatively strong signal. In contrast, a lower signal is needed to promote PC development in GC B cells which have spent more time in the germinal center and have collected more cumulative signals. This is supported by observations that the cytokine production patterns and expression of surface ligand of Tfh cells are altered over time during GC response. During type 2 immunological responses, late Tfh cells preferentially stimulate PC formation and express high levels of IL-4 and CD40L [54]. It is unknown whether variations in the proportion and phenotype of FOXP3+ T_reg_ in the GC could have an effect on the germinal center output.

## Factors affecting B cells selection for the memory pool

An important question to which we still do not know the definitive answer is, “What factors govern the fate of MBCs?” For decades, scientists have studied the development of MBCs and proposed several parameters that might play key roles in the fate determination of dividing B cells. Below, we review some of these factors and their roles in selecting B cells for the memory pool.

### BCR–antigen affinity

**Fig. 3:**
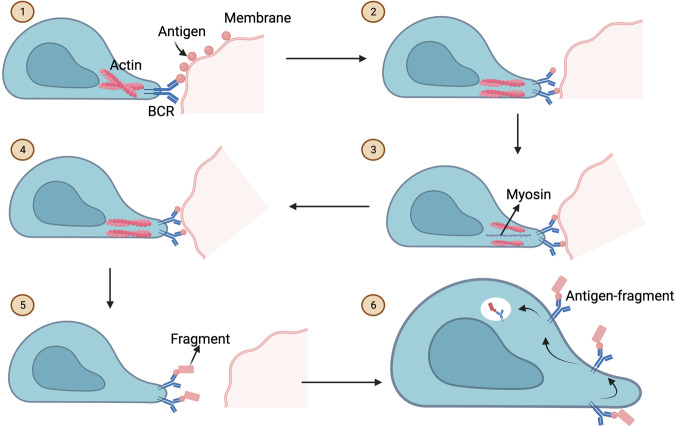
Antigen affinity selection by GC B cells. When exposed to an antigen (step 1), GC B cells produce distinct pod-like structures (rich in actin and ezrin) that concentrate BCRs (step 2). GC B cells utilize myosin-dependent processes to apply pressure to BCR–antigen complexes on the membrane of antigen presenting cells (FDCs) (step 3), thus deforming the antigen-bound membrane (steps 4) and, eventually, capturing the antigen and fragments of the associated membrane (step 5). The antigen and associated membrane fragment (antigen-fragment complexes) are then internalized for processing (step 6). High-affinity BCRs can withstand the pressure generated by the GC B cell on the BCR–antigen complex better than low-affinity BCRs, and as a result, high-affinity BCRs bind, process, and deliver more antigens to the T follicular helper cells.

BCR–antigen affinity studies have shown that the selection of B cells for the memory pool is linked to their antigen-binding affinity. Shinnakasu et al. [52] showed that B cells with a higher affinity for antigens develop into PCs, whereas cells with a lower affinity join the MBC compartment. Studies have shown that the ability of B lymphocytes to interact with antigens, internalize them, and process them for presentation is strongly correlated with their affinity for the antigen [55–57]. Currently, little is known about the precise mechanisms which B lymphocytes utilize to determine affinity towards soluble antigens. It has been proposed that B cells form a BCR–antigen complex on the cell membrane and exert pulling forces to capture the antigen. Higher-affinity interactions are more likely to successfully internalize the antigen and present it to the Tfh cells, because they have a higher ability to withstand pulling forces. This implies that the amount of antigen captured by B cells from follicular DCs (FDCs) for presentation to Tfh cells in the GC is an indirect determinant of B cell affinity [58] (Fig. 3).

Previously, it was believed that the differentiation of GC B cells into MBCs was a random naturally occurring process. This belief was largely based on studies in which anti-apoptotic factors, such as Bcl2, were overexpressed [59], which led to a significant rise in the low-affinity B cells in both the GC and MBC pools without having an impact on the selection of high-affinity PCs. Two recent investigations using monoectopic antigens (i.e., haptenated antigens and hen egg lysozyme antigens) have refuted this idea, demonstrating the presence of controlled instructions in the selection of GC B cells for the MBC compartment [52, 60]. First, although the low-affinity compartment of the LZ serves as the primary source of the MBC pool, high-affinity light zone GC cells also predominantly contribute to the memory cells pool. Second, the MBC pool is formed early in the immune response when B cells have acquired few somatic mutations, contributing, at least partially, to the cumulative accumulation of MBCs with relatively low BCR affinities. The latter is consistent with previous findings [51]. Furthermore, Brdu (5-bromodeoxyuridine)-pulse labeling enabled scientists to provide evidence that a temporal switch occurs in GC responses as they mature; they initially generate MBCs with fewer SHM and later produce long lived PCs with high SHM [51]. The lack of mutations in MBCs implies that B cells present less strict affinity-based selection and wider BCR cross-reactivity than the highly selective and mutated BCRs of long-lived PCs. This is supported by recent studies that have demonstrated that affinity-based selection is unequally applied to the precursors of MBCs and PCs, resulting in the generation of low-affinity, broadly reactive MBCs and high-affinity, highly selective long-lived PCs. Moreover, the analysis of antigen-specific MBCs and PCs provided evidence that Initial output response of GCs was highly selective PCs in response to immunization [61]. In contrast, only approximately 65% MBCs had the capability to produce high-affinity antibodies, which indicates their less-stringent selection. Concordantly, supplementary studies demonstrated that only the GC cells acquiring antigen-specific high-affinity BCRs via SHM differentiate into PCs [62]. All these studies strongly imply that antigen-affinity plays important role in the selection of GC B cells towards the memory pool.

### Cytokines, transcription factors, and signaling molecules

Our understanding about the transcription factors and signals that regulate differentiation of GC B cells has advanced significantly in recent years. The presence of functional and transcriptional differences at the early and late time points in the GC reaction suggests that several signaling molecules and transcription factors may be involved in fate determination. MBC-derived cytokines participate in the inflammatory milieu of viral infection [63]. Research suggests that the cytokine-induced transcription factors in B cells also control their behavior during primary response. For instance, the interferon -γ (IFNγ)-driven expression of T-bet (or TBX21) is a crucial factor for IgG2a class switching [64, 65]. Coincidentally, these transcription factors are required for Ig-specific MBCs. Compared to naïve B cells, IgA+ and IgG2a+ MBCs express higher levels of retinoic acid receptor-related orphan receptor-α and T-bet, respectively. More recently, Nicole et al. [66] demonstrated the critical role of Tfh cell-derived IFNγ in pulmonary immunity via the development of CXCR^3+^ resident MBCs in the lungs. These transcription factors are essential for the MBCs survival, likely because they regulate the transcription of genes encoding BCR components [67].

Regarding selection mechanisms, Shinnakasu et al. [52] demonstrated the favorable selection of Bach2-high light zone GC B cells into MBC compartment, along with the evidence of an inverse correlation between Bach2 expression and T cell help signatures. This suggests the presence of an affinity-dependent threshold for Tfh cells, below which differentiation towards the MBC pool is favored. This model suggests that low levels of Tfh cell help is sufficient to induce MBC generation; however, it is unable to drive PC differentiation and recycling to the DZ of the GC. Supporting this notion, Bannard et al. showed that mice with CXCR4-defecient B cells (unable to access the DZ) showed increased entry into the MBC compartment, which could be attributed to the “misdirected Tfh cell help.” In contrast, differentiation into PCs and the maintenance of GC, which require significantly increased levels of Tfh cells, were reduced in B cells lacking CXCR4 [50]. Similarly, increased MBC generation and a reduction in GC B cells have been observed in mice with IL-21R-deficient B cells [20, 49]. Because IL-9 is an inflammatory cytokine, the recently described Tfh-mediated role of IL-9 in MBC generation appears contradictory to the above-mentioned concepts. However, because IL-9 also supports the “immunosuppressive” functions of Treg cells [68], it may act via follicular regulatory T cells (Tfr) in the GCs. Alternatively, IL-9 may play a survival-promoting role as a bystander [69]. IL-9 receptor signaling has also been shown to regulate the humoral recall responses of MBCs [69], which suggests that the role of cytokines extends beyond the generation, and maintenance of B cell memory.

**Fig. 4:**
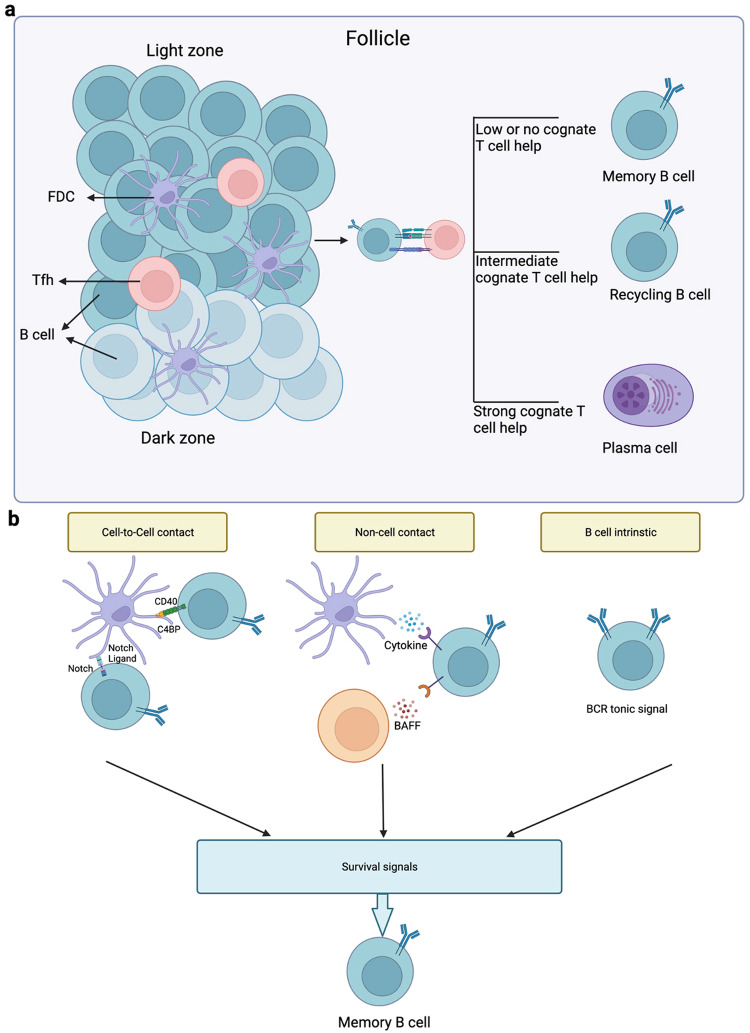
Overview of antigen affinity, signaling molecules, and survival factors in fate determination. **a** Once the B cell receptor (BCR) binds to the antigen presented by the follicular dendritic cell (FDC), memory B cell development depends on a low level or no T cell help being received by the germinal center (GC) B cell. An intermediate level of help from helper T cells (Tfh) predisposes the GC B cells to re-join the dark zone (DZ) by inducing c-Myc expression. Strong cognate T cell help favors the differentiation of GC B cells into PCs via IRF4+ c-Myc+ plasma precursors. **b** Among the non-cognate survival signals for MBC generation, follicular dendritic cells (FDCs) deliver signals to GC B cells via CD40–C4BP interactions and notch–notch ligand interactions. B cell-activating factor of the tumor-necrosis-factor family (BAFF) and cytokines also act as cell contact-independent signals in fate determination. BCR expression on the GC B cells could also offer survival signals via tonic cues. Reproduced with permission.

The significance of c-Myc expression in the proliferation of GC cells has been demonstrated, showing that nearly 10–30% of LZ GC B cells undergoing positive selection briefly present strong c-Myc expression. A previous study showed that Tfh cells strongly upregulate c-Myc expression in LZ GC B cells, which is suppressed in the DZ. Among the positively selected c-Myc+ LZ GC B cells, the functionally distinct IRF4+ c-Myc+ cells ( ~ 10%) [70, 71] had a higher BCR affinity than the IRF4− c-Myc+ cells, and preferentially headed toward the PC fate instead of recycling in the GC. More help from Tfh cells (especially CD40-dependent signaling) is required for the favored development of c-Myc+ IRF4+ cells than for that of c-Myc+ IRF4− cells. These PC precursors (c-Myc+ IRF4+ cells) exit GCs to complete their development into Blimp1+ early PCs [72, 73]. Thus, strength of Tfh cell help (defined by the duration of B cell– Tfh cell contact) determines the fate (PC precursor or recycling to GC) of LZ GC B cells that underwent positive selection [69] (Fig. 4a).

Recently, CCR6 expression, cell cycle quiescence, and reduced S1pr2 expression have been linked to the identification of MBC precursors in the GCs [60, 74, 75]. Another study showed that CCR6 uniquely marked MBC precursors in the GC LZ [60]. These MBC precursor cells are quiescent and somatically mutated, with enhanced survival and transcriptional resemblance to classic MBCs. Most likely, these precursor cells upregulate S1pr1 and EBI2 while reducing S1pr2 levels as a mechanism to exit the GC. The MBC precursors with these expression patterns were located close to the edge of the GC LZ. Thus, Laidlaw et al. [75] speculated that MBC differentiation is initiated in GC reactions, producing MBC precursors that exit the GC and complete their development in the niches they migrate to.

### Survival advantage

The survival advantage of antigen-experienced B cells has been linked to increased MBCs in previous studies. Deletion of the BIM protein (which is pro-apoptotic) or the upregulation of Bcl2 (or other anti-apoptotic proteins in this family) was shown to increase the MBC pool compared to GC B cells [38, 59]. Inoue and Kurosaki mentioned their unpublished data (in a review article [69]) where they selectively deleted BIM in activated B cells, which led to a drastic increase in MBCs compared with GC B cells (unpublished data). Given the studies showing improved survival ability and elevated Bcl2 levels in MBC precursors in the GC [60, 74, 75], the differentiation of LZ GC cells into MBC precursors is likely dependent on the acquisition of such survival capacity. As it is assumed that most GC B cells marked for apoptosis are low-affinity cells, the subject of “how low-affinity LZ GC B cells gain a survival advantage and become MBC precursors” appears to be one of the mysteries of MBC development.

According to a recent imaging-based study using new apoptosis reporter mouse, c-Myc+ LZ cells and some Nur7– cells (lacking BCR signals, and thus possibly expressing low-affinity BCRs) are shielded from apoptotic death [76]. Inoue et al. [69] suggested that apoptosis-resistant Nur77 cells could be memory precursor cells [69]. The GC microenvironment is believed to provide these cells with antigen-independent survival signals that may have specific molecular characteristics. For instance, the FDCs are believed to influence germinal center B cell survival through; i) the delivery of co-stimulatory signals via CD40- C4BP interactions, ii) notch–notch ligand interactions, and iii) cytokines [77]. Bystander delivery of the survival factor BAFF from Tfh cells is also possible [78]. Moreover, as an intrinsic factor of B cells, BCR expression alone “generating a tonic signal” could be a strong possibility in GC B cells [69] (Fig. 4b). These data imply that survival advantage plays a significant role in GCB cell fate determination.

### Pathogenic microenvironment

**Fig. 5:**
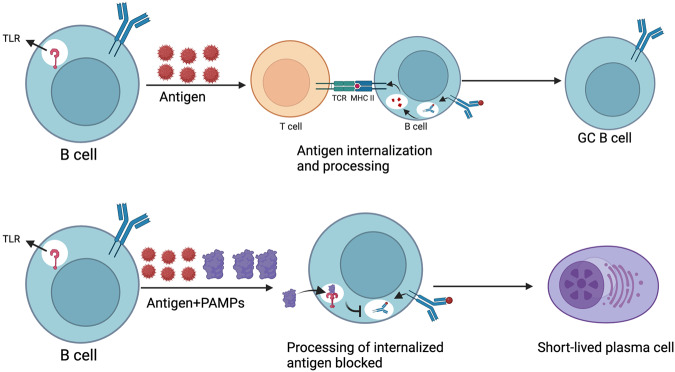
Effect of pathogens on fate determination of naïve B cells. Both BCRs (for antigen binding) and TLRs (which respond to PAMPs) are expressed on naïve B cells. When only the antigen is present, the B cells will internalize it, and process it, then present it on MHC- class II molecules. Interaction with preactivated, antigen-specific T cells will induce the proliferation and differentiation of antigen-activated B cells into germinal center (GC) B cells. In contrast, when the antigen is also accompanied by PAMPs, the TLR signaling inhibits the processing of internalized antigens and directs the B cell towards proliferation and development into short-lived PC. BCR B cell receptor, TCR T cell receptor, TLR Toll-like receptor, PAMPs Pathogen-associated molecular patterns. Reproduced with permission.

Scientists have begun to explore the impact of the microenvironment on the fate determination of naïve B cells. The presence of a pathogen itself or its products and the impending danger signals sent to the host via innate immune receptors constitute crucial environmental components. Recently, PAMPs, especially the CpG ligand for TLR9, were shown to unexpectedly block processing and presentation of the antigen after internalization, but before being sent to the antigen processing compartment [79]. As a result, B cells activated by CpG oligodeoxynucleotides have a lower ability to acquire T cells. Concurrent studies discovered that inability of activated B cells to get T cell help after BCR signaling led them to apoptosis, which has been attributed to their induced mitochondrial dysfunction serving as a “metabolic clock” [27]. These experiments further demonstrated that TLR9-stimulation promoted the proliferation and differentiation of antigen-activated B cells into low-affinity, short-lived PCs, while protecting them from apoptosis. Overall, these findings suggest a mechanism by which naïve B cells that are activated by pathogens or their products are predisposed to swift differentiation into short-lived PCs as opposed to participating in the “time-consuming” GC reactions (Fig. 5). Several studies have discussed the roles of the microenvironment (e.g., PAMPs or CpG) in immune responses and the fate determination of activated B cells. Thus, further evaluation of the precise role of the microenvironment in B cell fate determination will not only increase our understanding of several aspects of MBC development but also help in the development of vaccines with improved responses and protection against various diseases.

Response to booster shots, or viral reinfection has also been studied in the peripheral tissues. Influenza reinfection causes the development of tertiary lymphoid structures, which are sites of potential GC reactions and the production of resident memory B (B_rm_) cells and resident memory Tfh cells in the lungs [80]. A subpopulation of memory Tfh cells has been reported to reside long-term in the draining lymph nodes [81]. This prolonged residence of B_rm_ or memory Tfh cells may explain why a local antigen booster facilitates a recall GC responses better than distal [82]. After an initial influenza infection, antigen-specific CXCR3 + CCR6 + B cells and CXCR3 − CCR6 + bystander B_rm_ cells are generated in relation to B_rm_ cells [83]. This finding is not exclusive to influenza infections; and has also been linked to SARS-CoV-2 infections [84]. It is most likely a widespread occurrence in lung infections. The observations indicate that greater IgM Fc receptor expression, especially by bystander CXCR3 − CCR6 + resident memory B cells, may contribute to booster responses by binding IgM immune complexes, even if the precise role of bystander memory B cells is still unclear.

Alveolar macrophages play a pivotal role in triggering humoral recall responses in the event of influenza reinfection. They achieve this by secreting IFNγ and CXCR3 ligands (CXCL9/CXCL10), which in turn stimulate the recruitment of CXCR3 + resident memory B cells into foci of infected cells [85]. In the light of all these findings, Inoue and Kurosaki suggested that it is reasonable to assume that B_rm_ cells and resident memory Tfh cells are recruited close to alveolar macrophages, where these immune cells interact and exert protective immunity, even though it is unclear whether memory Tfh cells are necessary for the initiation of these recall responses [86].

### Metabolic reprogramming

Recently, the role of metabolic reprogramming during the transformation between effector cells (which are actively proliferating) and quiescent memory cells has gained more attention than the survival advantages of MBCs. Pioneering studies exploring metabolic reprogramming have been conducted on CD8 + T cells. Memory CD8 T-cells, unlike effector CD8 + T-cells (which perform glycolysis to produce NADH and ATP, have less mitochondrial mass: indicating anabolic metabolism), have a higher mitochondrial mass that support oxidation of mitochondrial fatty acids (catabolic metabolism) [87]. This equipped memory T cells with a bioenergetic advantage. The transition of cells from effector to memory phase occurs in a metabolically restricted environment where nutrients, growth factors and oxygen levels along with other signals are limited. Under these conditions, reduced ATP levels trigger AMPK activation, thereby suppressing anabolic pathways and promoting catabolic pathways. Supporting this, metformin induces the activation of AMPK (which is a potent activator of autophagy) and increases CD8+ memory T cell generation [88]. Given the important role of autophagy in the generation and survival of MBCs [89], it is likely that metabolic reprogramming occurs during MBC development in the contraction phase of the effector response [89]. This may explain the accumulation of IgG+ MBCs and the reduction in GC cells in IL-21 or IL-21R deficient mice [49]. GC B cells rely on glycolysis for maintenance; therefore, high cytokine levels are required as growth factors. Assuming that, similar to T cells, metabolic reprogramming to catabolic metabolism from anabolic metabolism occurs for B Cells as well, MBCs may not rely on cytokine levels for maintenance.

Questions have arisen regarding the main effector that is responsible for the contribution of low Tfh cell help in promoting MBC differentiation. In recent years, scientists have provided evidence for metabolic involvement in fate determination and survival. Pollizzi et al. [90] showed that asymmetric mTORC1 inheritance in dividing CD8 + T cells affects metabolic fitness, where daughter cells with low mTORC1 levels showed improved long-term survival in vivo. These results support the notion that memory CD8 + T cells decrease activity of mTORC1 favoring metabolic regulators like AMPK (known to suppress the activity of mTORC1), which can aid lymphocytes in managing stress and metabolic resources for prolonged survival [91]. Given the significance of low mTORC1 in the maturation of memory CD8 + T cells, it would be logical to assume that similar metabolic regulation (low mTORC1 activity) may also be essential to the development of MBCs in GC reactions [91]. It is likely that in the pre-GC stage, each activated B cell has varying degrees of mTORC1 activity, and low mTORC1 activity encourages the cells towards the memory fate. A recent study provided evidence in support of this theory by demonstrating that activated B cells containing greater AMPK levels avoid development into IRF4-high PCs and then join the quiescent memory pool [92]. To this end, it has recently been shown that mTORC1 activity in LZ GC B cells is closely linked to the level of Tfh cell help acquired [93], supporting the notion that MBC differentiation is more likely to be favored by low Tfh cell help.

### Transcriptional regulation of MBC differentiation

Laidlaw and Cyster [45] have fully reviewed the regulation of GC B cell fate at the transcriptional level. Here, we provide a brief overview of this information. As discussed above, the failure of low-affinity LZ GC B cells to acquire T cell help results in apoptosis [76, 94]. GC B cells that acquire less T cell help present lower mTORC1 activation and decreased biomass compared to those that have acquired more T cell help [93]. Moreover, the failure of these cells to express MYC affects both their cell division ability and the entry into the cell cycle [95]. MYC works in conjugation with MIZ1, promotes the development of PCs, and restricts their differentiation into MBCs [96]. These low-affinity GC B cells also have elevated BACH2 expression, presumably due to decreased transcriptional repression by mTORC1 [97, 98]. The expression of BACH2 promotes the differentiation of GC B cells into MBCs [52]. Although the exact mechanism responsible for BACH2-induced MBC differentiation is yet to be revealed, it may involve the downregulation of Cdkn1a/Cdkn2a genes, and the expression of the anti-apoptotic Bcl2l1 gene [99]. As reported previously [38, 100], the upregulation of survival promoting genes, like Bcl2, and the inhibition of pro-apoptotic genes aids the differentiation into memory B cells. All these studies point to a scenario in which low T cell numbers aid memory B cell differentiation by preventing mTORC1- and MYC-induced proliferation and the cell cycle progression, which favors survival of cell.

Both human and mouse GCs contain precursor memory (pre-Mem) B cells [60, 74, 75]. Despite exhibiting surface markers, GC B cells are usually located close to the edge of the LZ and resemble MBCs in terms of their transcriptional and functional characteristics. The mature GC B cells that are transitioning into MBCs are known as preMem B cells. They have recently undergone cell division and have had somatic mutations. Pre-Mem B cells likely need to leave the antigen-rich LZ to completely differentiate into MBCs. Thus, exiting the GC is facilitated by BCL-6 downregulation. BCL-6 promotes the expression of S1PR2, a germinal center-confinement factor, and inhibits the expression of pro-migratory receptors, such as EBI2 and S1PR1, which likely help in exiting the GC [101]. Moreover, BCL-6 induces the apoptosis of GC B cells by repressing the expression of BCL-2 [102]. Thus, the pre-Mem B gene signature is primarily driven by the loss of the BCL-6 transcriptional program [75]. Taken together, these results strongly support the importance and involvement of transcriptional regulation in GC B cell differentiation and fate determination.

## Heterogeneity of MBCs

**Fig. 6:**
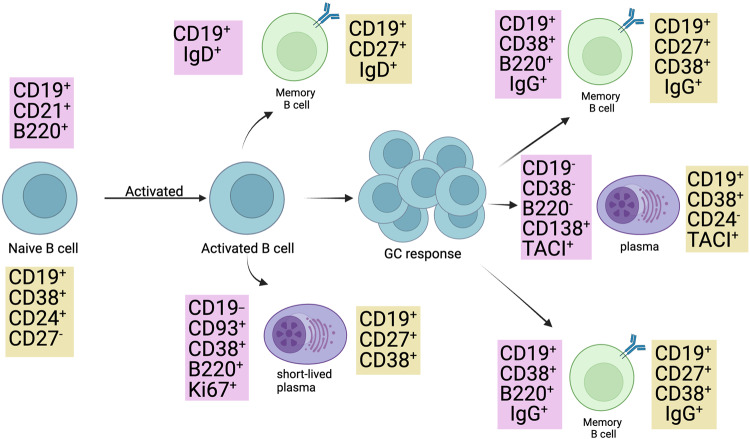
Cell surface markers of B cells and subtypes. Diagrammatic representation of the different fates of naïve B cells upon activation. The different cell surface markers expressed by activated B cells during proliferation are shown. Cell surface markers in purple boxes are expressed in mice, while yellow boxes contain the surface markers of different types of B cells in humans.

During a primary immune response, different classes of MBCs are produced in a spatiotemporal manner, implying that these MBCs serve different purposes [103]. With the advancement of labeling methods for identifying various antigen-exposed B cell types [10, 104] it has become possible to functionally identify and characterize the various MBC subtypes. Researchers have identified several surface markers that can be used alone or in combination to mark particular B cells or MBCs in both humans and mice [105]. Figure 6 depicts the various cell surface markers routinely employed in flow cytometry to identify B cells and MBCs. According to advanced research, the origin, function, and lifetime of MBCs differ among the different subtypes. This prompted investigations into the mechanisms underlying this heterogeneity and whether distinct MBCs subtypes are coordinately activated after a particular response. An observable paucity of IgE+ MBCs [106–108] necessitates reconsideration of the common notion that a certain class of immunoglobulin-expressing MBC responds to that class’s secondary memory responses. The reactivation of MBCs upon secondary challenge with pathogens is discussed in detail in the “Reactivation/secondary infection” sub section below.

While discussing the heterogeneity of MBCs, it is important to mention the resident memory B (B_rm_) cells (B_rm)_ [109], which establish, in part, by antigen encounter at the site of their residence. There is now clear evidence about their existence as well as their protective functions in peripheral tissues [110]. These cells are maintained in the barrier tissues and mostly positioned close to the possible re-infection site [111]. Currently, there is no definitive marker to distinguish tissue-specific B_rm_ cells from other MBCs subtypes. Thus, these cells are mostly identified on the basis of their functional properties. One way is the use of intravascular staining, a few minutes before euthanasia and tissue collection. This involves the intravenous administration of a fluorochrome-conjugated antibody specific for the population in question [110, 112]. Another definitive method to show a tissue-resident cell population is “parabiosis” [113], which connects the peripheral, but not lymphatic circulation, thus allowing the circulating cells to move freely between the parabionts. Sometimes, intravenous antibody infusion is combined with parabiosis to distinguish the true resident cells, from the circulating cells which have entered the tissues [114]. While our understanding about the B_rm_ is advancing, there remain fundamental knowledge gaps which limit our ability to target these cells in treating diseases, establishing tissue homeostasis, and preventing re-infections. Future studies may focus on elucidating the signaling mechanisms that govern the development and functional properties of B_rm,_ which may in turn inform “why certain sites, such as the female reproductive tract, do not appear permissive for the establishment of B_RM_ cells [115] ”.

## Reactivation/secondary responses

**Fig. 7:**
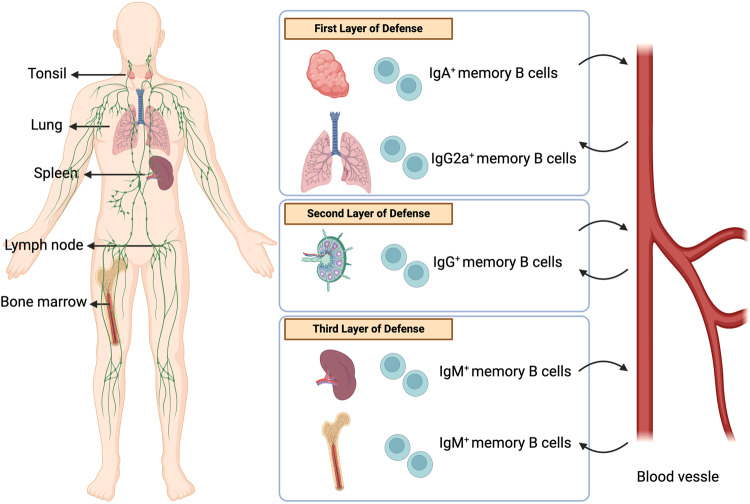
Heterogeneity in the memory B cell pool offers multilayered defense upon reinfection. Once generated, the immune system dispatches various types of MBCs with unique purposes throughout the body. The figure illustrates the placement of these functionally specific MBCs in different anatomical locations to constitute multilayered protection against reinfection. Aside from these strategically positioned “sentinel” MBCs, “patrolling” MBCs recirculate and serially monitor the secondary lymphoid organs for antigen. Reproduced with permission.

The reactivation of MBCs generated during the primary immune response is an area of active research. Once generated, MBCs must maximize their chances of encountering invading pathogens to offer efficient, long-lasting humoral immunity. The immune system accomplishes this by producing numerous types of MBCs, which are then deployed to various locations in the body to serve different functions [69] (Fig. 7). Upon antigen rechallenge (in secondary responses), MBCs provide multiple lines of defense against pathogens by either quickly differentiating into PCs or entering GCs to undergo several cycles of proliferation, somatic hypermutation, and selection. The efficiency of the recall response depends on the rapid and high production of class-switched antibodies upon antigen re-encounter in secondary responses. The IgA+ MBCs present on exposed mucocutaneous surfaces [116] form the first line of defense against invading pathogens. T-bet-induced IgG2a+ MBCs, which develop in response to viral infections, also reside in infection sites [67], such as the lungs [30, 117], and participate in recall responses. The presence of IgG+ MBCs in the draining lymph nodes provides a second line of defense against pathogens that breach the skin [81]. If the invading pathogens escape the skin and lymph node barriers, a third defense line is established by the IgM+ MBCs expressing FOXP1 [118] that reside in the bone marrow [119] and spleen [120, 121]. Studies have proposed that sentinel MBCs mainly develop in GCs in SLOs and then translocate to their respective niches, directed by various chemokine receptors (such as EBI2, CCR6, CCR7, CXCR3, CXCR4, and CXCR5 [67, 117, 122, 123]) that are generally associated with the positioning of T and B cells.

The generation of different types of MBCs in the primary responses suggests that they have different fates upon antigen re-encounter. Some MBCs favor differentiation into PCs, whereas others develop into GCs and undergo several rounds of proliferation and SHM to give rise to more efficient, high-affinity class-switched MBCs and antibody-secreting PCs. Several studies have proposed different rules for predicting the fate of MBCs during recall responses. A study conducted by Dogan et al. [10] showed that adoptively transferred IgM+ MBCs differentiate into mGC cells, whereas IgG+ MBCs primarily generate mPCs upon receiving a boost injection of sheep red blood cells. Subsequently, during an early recall response (challenge on day 320 after primary immunization), IgG+ MBCs predominated over IgM+ MBCs, preferentially differentiating into antibody-secreting cells [11]. The low response of IgM+ MBCs was attributed to circulating antibodies that outcompeted the low-affinity BCR on IgM+ MBCs for access to antigens. The adoptively transferred IgM+ MBCs differentiated into GC B cells upon antigen encounter and entered further rounds of class switching and affinity maturation to produce a high-affinity IgG response. In contrast, when a recall challenge was performed at a later time point, at which time the levels of circulating antibody titers (anti-specific IgG) and IgG+ MBCs were significantly lower, it resulted in the differentiation of IgM+ MBCs to GC B cells as primary responders.

Recent studies have elucidated the fate-predictive phenotype of MBCs. Schlomchik et al. [6, 51, 124, 125] reported that MBCs expressing CD80, CD73, and PD-L2 mostly give rise to PCs, whereas MBCs lacking these markers develop into GC cells, irrespective of their IgG or IgM expression. Furthermore, their research revealed that most IgG+ MBCs express CD73 and CD80, whereas IgM+ cells lack these markers. This is consistent with the observations of Pape et al. [11], who suggested that protective immunity against rapidly evolving pathogens requires MBCs to develop GC cells that further diversify their BCRs. Subsequently, McHeyzer-Williams et al. [126, 127] showed that a boost resulted in the differentiation of IgM+ MBCs into PCs, whereas IgG+ MBCs favored GC development and underwent further BCR diversification. Taken together, these studies suggest that both IgG+ and IgM+ MBCs have different propensities towards GC or PC differentiation in a recall response based on their BCR isotype, antigen affinity, and differentiation state. Recent research has shown that the fate of B cells can also depend on their location and access to Tfh cells. Resident memory Tfh cells located near MBCs in B cell follicles encouraged PC differentiation, while circulating memory Tfh cells favored the development of secondary GCs [128]. Moreover, these variables dictated antibody production kinetics as well as the functional characteristics of the secreted antibodies during the recall response. Studies have shown the importance of highly selective, high-affinity antibodies synthesized by long-lived PCs against a homologous (pathogen) challenge, while highlighting the role of MBCs against variant pathogens that are poorly neutralized by these antibodies [129]. The antibodies produced by MBC-derived PCs can neutralize both the wild-type and variant strains of a pathogen equally, or even neutralize the variant virus more effectively [129–131].

Variant proteins largely trigger the IgM+ MBCs with the most varied and least altered V genes, according to the immune responses of mice challenged with the same or variant viral proteins after injection with dengue virus envelope proteins [132]. These findings strongly support the notion that populations of highly diversified MBCs are essential for offering widespread defense against infections by various viruses. Finding a method of selectively activating these MBC populations is a future challenge.

## Interactions with and dependence on other cell types

While virus-specific MBC activation and robust differentiation to PCs is possible without T cell assistance [133], it is widely accepted that the activation of monomeric protein antigen-specific MBCs requires the collaborative assistance of T cells [104, 119], prompting research into the responsible memory T cells. The CXCR5-expressing CD4+ memory T cells found in the T cell zone near the B cell follicle have a Tfh phenotype and assist B cells. Most conventional markers of effector Tfh cells (e.g., BCL-6 and PD1) are downregulated in memory Tfh cells, although they are swiftly upregulated upon re-stimulation [134–137]. The low but still obvious expression of CXCR5 in memory Tfh cells distinguishes them from other subtypes of T cells memory. Memory Tfh cells in the spleen or lymph nodes rise in the T cell zone, at the B cell-T cell interface, and in B cell follicles as a result of CXCR5 expression [134, 138]. The T cell–B cell border and follicles are the preferred habitat of CXCR5+ memory Tfh cells [134]. Unlike naïve T cells (which can differentiate into Th1, Th2, Th17, and Treg cells), Tfh cells can be retained as CXCR5+ memory Tfh cells for an extended period and differentiate preferentially into Tfh cells upon activation [138]. Recently, memory Tfh cell loss was found to prevent MBCs from reactivating and dividing into PCs [134], demonstrating the importance of memory Tfh cells in successful recall antibody responses. To explore the activation of memory Tfh cells and their interaction with and contribution towards MBC activation, Ise et al. [134] adoptively transferred CXCR5+ memory Tfh cells into naïve mice and found that they home the border of T cell–B cell zone and the follicles in spleen within 24 h. These memory Tfh cells are capable to live for extended durations and are quickly reactivated when antigen recall occurs, upregulating Bcl6 and transforming the cells into Tfh effector cells. This phase is dependent on the presentation of MHC-II peptides on MBCs, but not those on normal DCs, implying that MBCs could directly activate memory Tfh cells in the follicle. Memory Tfh cells may be directly activated by MBCs in extrafollicular areas such as bone marrow, where dedicated DCs and other antigen-presenting cells may be uncommon.

FDCs are hypothesized to play a role in the preservation of MBCs and subsequent recall responses, in addition to memory T cells [139]. Although it is widely accepted that FDCs retain antigens for extended periods, the functional relevance of antigen persistence in the memory responses is controversial, and the underlying mechanism of the FDCs’ role in MBCs’ maintenance is unknown. FDCs cycle CR1-bound complement C3d-coated immune complexes in non-degrading endosomal compartments, thus preventing the antigen from being destroyed and allowing it to be available to B cells for longer [140]. This mechanism may play a role in the persistence of GCs as well as in the formation and/or maintenance of MBCs. Because exogenous protein molecules, particulate matter, and the invading pathogens are known to be quickly transported to FDCs, FDCs may contribute by quickly presenting antigens during a recall response. This transport is sped up by their binding to the pre-existing antibodies and subsequently, complementary activation occurs. Because IgG1+ MBCs are found near constricted GCs, which include FDCs in close proximity, these MBCs are more likely to collect secondary antigens [104].

## B cell strategies against infection

### Permissive selection in GCs

MBCs and their subsequent antibodies are of prime importance in preventing viral infections. In contrast, highly mutagenic viruses such as influenza and HIV continue to elude the immune system. Influenza viruses seasonally gain “escape mutations”, whereas HIV‐1 keeps mutating at a rapid pace in people with chronic infection. Such mutational process leads to the evolution of variable and conserved regions of the envelope proteins, which are the main targets for antibody-mediated protection. The conserved regions contain epitopes for widespread reactive antibodies. Viruses frequently use two methods to avoid antibody detection of their conserved domains. First, because they offer steric hindrance that blocks antibody access, the conserved domains have weak immunogenicity or are immunologically subdominant [141]. Second, epitopes inside the conserved envelope regions that structurally mirror host self-antigens control immunogenicity through immunological tolerance and reduce the formation of broadly reactive antibodies. Despite these challenges, the ability of GCs to gradually develop ex_t_ensively reactive BCR repertoires raises several questions. According to two recent studies, when germinal center reactions occur in the presence of polyepitopic native-antigens or viral infections, there is intraclonal competition between somatically hypermutated variants of the same clone that are particular to the same epitope, as well as interclonal competition between clones bearing different V(D)J arrangements [142, 143]. Furthermore, these studies showed that B cells with significantly varying affinities can coexist in the same GC, resulting in diversity and implying that GC selection may be less rigorous than previously assumed. Moreover, Kuraoka and colleagues discovered that non-(antigen)-specific GC B cells could develop [142]. They postulated that these germinal center B cells identify in vivo-modified antigens, including cryptic epitopes revealed by degradation or the neoepitopes established by complement fixation.

The findings described above provide useful insights for the development of widely reactive GC B cells. First, GCs enable B cells that are few at first and/or express low-affinity BCR to continue to be viable and stay within the GCs, even in the midst of usual affinity maturation. Second, structural modifications of complex protein antigens can alter the balance between host antigen structural mimicry and epitope immunogenicity. Citrullination (covalent modification) of the host protein epitope causes autoimmunity and breaks self-tolerance in rheumatoid arthritis [144]. It should be noted, during viral replication, viral envelope proteins can experience significant structural modifications [145, 146], which may explain the significant overexpression of broadly reactive B cells in lungs GCs compared to spleen GCs in mouse models of influenza virus infection [147]. In addition, the infection-driven immunological milieu may weaken the immunological tolerance mechanisms which prevent the antigen from driving the selection of GC B cells which have autoreactive or polyreactive BCRs, in addition to the structural characteristics of the antigen. In autoimmune animal models, it has even been proposed that B cell intrinsic signaling via IFN‐γ affects GC B cell tolerance [148].

### Broadly reactive germinal center B cells are recruited into the memory pool

**Fig. 8:**
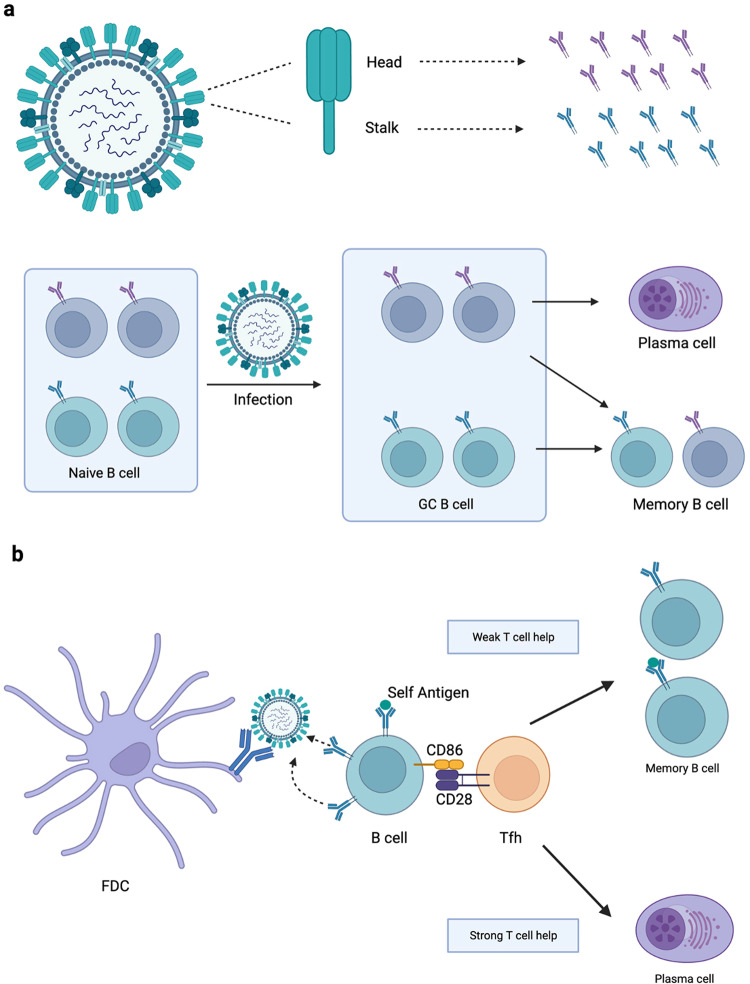
Generation of memory B cells upon viral infection. **a** Diagram illustration of the MBCs’ generation upon viral (influenza) infection. Displayed in purple are the antibodies against immunodominant viral epitopes of the influenza virus (anti-HA head domain), while the green antibodies are against the immunologically subdominant conserved viral epitopes (anti-HA stalk domain). **b** Broadly reactive low affinity GC B cells are more likely to join the MBC pool. Autoreactive germinal center B cells are thought to have a lower affinity for both self-antigens and foreign antigens and express less CD86, resulting in reduced Tfh cell assistance and joining the memory B cells pool. Reproduced with permission.

Following the development of broadly reactive germinal center B cells, they are often selected for the memory compartment instead of the long-lived PC compartment [149, 150] (Fig. 8a). There are two possible approaches here. First, many broadly reactive GC B cells, although not all of them, have low affinity (which was previously mentioned in the context of the basic mono-epitope system), making them more likely to be selected into the memory pool. Second, there are relevant implications from the research of Sabouri et al. [151]. They demonstrated that autoreactive GC B cells first gain mutations which lower their affinity towards foreign antigen as well as the self-antigen. The resulting low-affinity GC cells are then more positively selected because they have less self-antigen occupying their BCRs, making them better capable to identify the foreign antigen in vivo. Considering that these GC B cells retain certain anergic B cell features as a result of persistent attachment to self-antigen [152], they are expected to express lower surface BCR level and are unable to elevate CD86, which is a known co-stimulatory receptor of T cell activation [69, 152, 153] (Fig. 8b). Therefore, these autoreactive or poly‐reactive GC B cells have a reduced ability to activate Tfh cells, which reduces their level of assistance and facilitates their recruitment into the MBC compartment.

### Prophylactic antibodies

Although vaccination is the most efficient and affordable method of preventing infectious diseases to elicit B cell memory, the development and production of efficient vaccines for many of the world’s deadliest infections, such as AIDS (acquired immunodeficiency syndrome) and malaria, has proven challenging. Thus far, there has been little success. Owing to the lack of success in vaccine development for these two diseases, along with many others, initiatives were introduced to avoid efforts to develop vaccines and instead to provide extremely efficient broadly neutralizing antibodies as preventatives (discussed by Walker and Cockburn before [154, 155]). The idea is not entirely new because passively transmitted antibodies were used to treat several infectious diseases, including tetanus and diphtheria, and later for several viral diseases, including hepatitis B virus, hepatitis C virus, and respiratory syncytial virus infections. The novelty, however, lies in the efficient production of unique, highly effective, and broadly cross-reactive human mAbs against a variety of viral infections, such as HIV and parasitic malarial infections. Moreover, the cross-reactivity, of these antibodies along with their potency, and half-life can be improved through antibody engineering. A thorough evaluation of donors and then selection with the desired serum antibody profiles, along with the advancement of high-throughput human B cells isolation techniques are essential for the identification of these unique human antibodies. Currently, passive antibody prophylaxis is a potential substitute for HIV vaccines. Moreover, a different method of antibody prophylaxis may be possible after recent breakthroughs in the vector-mediated antibody transfer in macaque and mouse, wherein a single injection enables the sustained antibodies delivery [23, 24]. The outcomes of clinical trials for the development of monoclonal antibodies (antiviral and antimalarial) will determine the success of this strategy in the near future.

## Perspectives

Significant progress has been made in delineating the cellular and molecular processes involved in the development and responses of MBCs. Recent advances in immunobiology have generated novel insights for an improved understanding of the innate and humoral immunity provided by long-lived MBCs in various diseases. A flurry of papers from different groups, describing the results of studies conducted on transgenic and knockout mice, have indicated several important cellular/molecular factors (including cytokines and transcription factors) involved in the generation and maintenance of MBCs, which could prove beneficial in the development of vaccines against various diseases.

Several studies have suggested that the development of MBCs into plasmablasts or GC B cells upon reactivation is compartmentalized into various sub-populations; to benefit from this compartmentalization of MBC function in vaccine design and development, researchers would need to better understand the overall mechanisms that drive the differentiation of these MBC subsets. Moreover, a plethora of studies has provided evidence supporting disease-specific memory. In addition to rapidly differentiating into high-affinity antibody-secreting plasmablasts, reactivated MBCs also produce Th1 cytokines like IL-12 and IFNγ, and aid in antigen presentation to CD4+ and CD8 + T cells, all of which helps to facilitate disease-specific cell-mediated immune responses. Thus, quantifying “pathogen-specific” differences in the induction of transcriptional and metabolic signatures in B cells could provide further insight into disease-specific immune responses.

As with most scientific achievements, these discoveries are accompanied by technological innovations, including single-cell RNA sequencing and newer techniques, such as Chip-Seq, spatial transcriptomics, and improved algorithms for the study and analysis of post-genomic processes (via transcriptional, proteomic, and metabolomic data). Currently, with diseases such as coronavirus disease 2019, there are opportunities to apply these approaches, combined with existing data, to identify novel targets in vaccine development that could elicit enhanced B cell memory responses and compare them with the knowledge accumulated by previous research on other pathogens, vaccines, and model immunizations. Incorporating advanced technologies would also help to delineate the metabolic requirements of B cells in vivo and at the single-cell resolution. Nonetheless, the therapeutic use of MBCs is limited by several knowledge gaps that must be addressed in future studies. Future research aiming to further understand the molecular and cellular mechanisms of B cell-specific immunological memory in human and animal diseases will set the stage for the discovery and establishment of advanced approaches and tools in immunodiagnostics and improved vaccine development. Cross-comparative studies in humans and mice will be helpful for defining the conserved properties (transcriptional and metabolic) of B cells. In the long run, these studies will hopefully also clarify the key rules for effective versus ineffective immune responses, allowing the rational construction of new vaccines and therapeutic interventions against autoimmunity and allergies.
